# Virtual Reality and Physiotherapy in Post-Stroke Functional Re-Education of the Lower Extremity: A Controlled Clinical Trial on a New Approach

**DOI:** 10.3390/jpm11111210

**Published:** 2021-11-16

**Authors:** Carlos Luque-Moreno, Pawel Kiper, Ignacio Solís-Marcos, Michela Agostini, Andrea Polli, Andrea Turolla, Angel Oliva-Pascual-Vaca

**Affiliations:** 1 Department of Physiotherapy, University of Seville, 41009 Seville, Spain; angeloliva@us.es; 2 Laboratory of Neurorehabilitation Technologies, San Camillo IRCCS, 30126 Venezia, Italy; andrea.turolla@hsancamillo.it; 3 Physical Medicine and Rehabilitation Unit, Azienda ULSS 3 Serenissima, 30126 Venice, Italy; pawelkiper@hotmail.com; 4 Unit of Humans in the Transport System, Swedish National Road and Transport Research Institute (VTI), Linköping University, 58330 Linköping, Sweden; ignacio.solis@vti.se; 5 Department of Neuroscience, Section of Rehabilitation, University-General Hospital of Padova, 35128 Padova, Italy; michela.agostini@unipd.it; 6 Pain in Motion International Research Group, Department of Physiotherapy, Vrije University Brussel, 1050 Brussel, Belgium; andrea.polli@vub.be

**Keywords:** physical therapy modalities, virtual reality, stroke, gait disorders, neurologic, postural balance

## Abstract

Numerous Virtual Reality (VR) systems address post-stroke functional recovery of the lower extremity (LE), most of them with low early applicability due to the gait autonomy they require. The aim of the present study was to evaluate the feasibility of a specific VR treatment and its clinical effect on LE functionality, gait, balance, and trunk control post-stroke. A controlled, prospective, clinical trial was carried out with 20 stroke patients, who were divided into two groups: the first group (VR + CP; *n* = 10) received combined therapy of 1 h VR and 1 h of conventional physiotherapy (CP) and the second group (CP; *n* = 10) received 2 h of CP (5 days/week, for 3 weeks). The following pre-post-intervention measuring scales were used: Functional Ambulatory Scale (FAC), Functional Independence Measure (FIM), Fugl-Meyer Assessment (FM), Berg Balance Scale (BBS), and Trunk Control Test (TCT). Only VR + CP showed a significant improvement in FAC. In FIM, CP presented a tendency to significance, whereas VR + CP showed significance. Both groups improved significantly in FM (especially in amplitude/pain in VR + CP and in sensitivity in CP) and in BBS. In TCT, there was a non-significant improvement in both groups. The results indicate that the intervention with VR is a feasible treatment in the post-stroke functional re-education of the LE, with the potential to be an optimal complement of CP.

## 1. Introduction

Stroke is one of the most serious neurological disorders, classified as the second cause of death in the world [1,2], with approximately 17 million new diagnoses every year [2]. Although the global rate of stroke or cerebrovascular accident (CVA) is decreasing, the rates observed in young adults are increasing, and the absolute number of cases in this population is expected to increase sharply in the next years (for 2025, 1.5 million Europeans will suffer a CVA every year) [3]. After a stroke, 75% of survivors acquire some type of disability (from mild to severe). In cases of moderate stroke, functionality improves during the first 3 months after stroke, and then it decreases significantly [4], which is why a long-term physiotherapeutic treatment is required. Thus, at 3 months, there is still a considerable margin for improvement in all functional measures: 85% of patients still have difficulties in the gait, 78% have not acquired specific norms for upper extremity function, and 29% still show balance impairment [5]. Of all the deficits presented by CVA patients, motor coordination of the paretic LE seems to explain better the limitations in the execution of different functional activities that involve the LEs [6]. Gait control has been described through a tripartite model consisting of steeping (basic reciprocal rhythmic movements of the LEs), balance, and adaptability (adaptation to the task and environmental demands). Among these components, dynamic balance can predict falls in this type of patient more accurately than gait speed [7]. On its part, adaptability (often greatly compromised after stroke) is crucial for safe walking in the household and in the community [8]. The trunk plays a fundamental role since there is a strong relationship between trunk control, balance, and mobility when sitting and walking. Thus it would be beneficial to include a specific action in the physiotherapeutic programme [9]. At the functional level, hemiparetic gait usually involves a decrease of trunk acceleration, whereas instability and asymmetry increase due to the lower movement toward the paretic side [10]. However, it is important to identify whether the deviations from normality in the functional parameters after stroke are a direct result of CVA or learned or adapted compensations to fill these deficits [11], since the physiotherapeutic approach will be different in each case. 

The provision of integrated programmes of functional recovery with adequate resources, doses, and duration is essential in the treatment of CVA [12]. Intensive physiotherapy is decisive for the improvement of the motor function after stroke, which potentially promotes neuroplasticity to learn new motor skills [13,14], being especially effective when increased at least 16 h in the first 6 months after the CVA [15]. However, post-stroke neuroplasticity also occurs in chronic patients [16], with the repeated practice of specific tasks during the acquisition of motor skills being fundamental to increase dendritic growth and the strength and number of synapses [17]. Moreover, the literature shows that training with repetitive tasks improves LE functionality after stroke, prevailing up to 6 months after the treatment [18]. Despite the proven efficacy of a combined approach of conventional physiotherapy (CP) techniques in the improvement of LE functionality after stroke, there is not enough evidence to conclude that one approach is more effective than another [19]. Some modalities of physiotherapy have proved their effectiveness, such as neuromuscular electrical stimulation [20] and sensory retraining [21]. There is also evidence (although limited) on the effectiveness of therapeutic exercise, training with repetitive tasks, motor training, virtual reality (VR), and the use of unstable platforms in the improvement of balance after stroke [22].

There are tools, such as robotics, that complement post-stroke physiotherapy [23,24]. Some of them are widely used in clinical practice (e.g., the treadmill for gait re-education), and they seem to have a short-term effect on gait speed and endurance, although only in patients who could walk autonomously at the beginning of the treatment [25]. Moreover, contact with the floor/ground seems to be a determinant in parameters such as step symmetry [26]. Thus, it is necessary to validate tools that enable early intervention, even in patients with reduced walking capacity at the beginning of the treatment, in order to start re-educating symmetric patterns that will be established in the long-term. To achieve this gait symmetry, the administration of visual and proprioceptive feedback seems to be key, including the presentation of coherent information in a correct kinematic manner [27,28]; this allows the patient to progressively improve his/her movement quality, approaching normality, especially in patients who present numerous compensations with the non-paretic hemi-body [29]. The VR intervention is adjusted to the general recommendations for post-stroke motor recovery, which must be based on the practice of specific repetitive tasks (with the already mentioned advantages that this practice contributes to motor learning) and high-intensity tasks with performance feedback [30,31,32].

One of the main difficulties in this intervention is the broad definition of VR [33], which comprises a large amount of systems with great heterogeneity. One of the characteristics that define this type of system is the selection of a series of parameters (e.g., the degree of immersion, type of feedback, etc.), on which the treatment appears to be more effective in patients with specific neurological pathologies, depending, among other aspects, on which sensory channels are most affected. Moreover, VR systems can be classified according to the purpose of their hardware into two types: VR systems created specifically for therapeutic purposes (they incorporate principles of neurorehabilitation that potentially improve learning and functional recovery) and commercial VR videogame systems (mainly designed for leisure) [30]. The literature shows that, although these interventions are not an alternative to CP, VR can be beneficial for the improvement of functionality when used as a complement of CP [33]. However, despite promising results in the functional recovery of the LE [34,35,36,37,38,39], the evidence on their effectiveness in balance and gait is still very limited [33]. The conclusions of the referenced studies show the difficulty of establishing treatment protocols in patients who mostly present very individualised deficits after stroke. In addition, the design of the studies is very heterogeneous, as well as the intervention methods (treatment intensity, characteristics of the patients, systems used, and additional interventions performed in the patients). In addition, many of the systems used required patients to walk autonomously to be included, which limits the early initiation of VR in acute patients who are more functionally affected. Regarding balance recovery, the effect of VR is even less clear in patients with acute or subacute CVA [40]. Some studies have already shown that this type of intervention promotes recovery through the principles of motor learning and neural plasticity [41]; other studies have demonstrated the effectiveness of using a specific VR system applied to the functional improvement of the upper extremity (UE) after stroke [32,42,43] and its impact on the activities of daily living. The present study is a controlled clinical trial that proposes the adaptation of this system to the functional recovery to the LE in CVA patients. Our aim was to evaluate the feasibility and clinical effect of a treatment based on a specific VR system, administered in conditions of hospital routine in combination with CP, on the functionality of the LE in CVA patients. In addition, our specific objectives were to analyse the effects of this system on motor function, gait function, balance recovery, and post-stroke trunk control. 

## 2. Materials and Methods

Type of study: pragmatic, prospective, controlled, clinical trial [44,45,46].

### 2.1. Patients

The sample of patients diagnosed with CVA considered for this study was extracted from patients admitted to a hospital specialised in neurorehabilitation. A total of 20 patients (demographic and individual data presented in Table 1) participated in the study, divided into 2 groups: VR + CP and CP.

The inclusion criteria in the selection of participants were the diagnosis of a first stroke (ischemic or haemorrhagic). The exclusion criteria considered the following conditions: clinical evidence of cognitive deterioration (score below 26 points in the *Mini-Mental State Examination* (MMSE)) [47], history of traumatic brain injury (TBI) (e.g., cranioencephalic traumatism, etc.), findings of verbal comprehension deficit and/or apraxia (score below 62 points in the *De Renzi* test) [48]. When it was not possible to determine the cognitive state of the patient using the MMSE (e.g., due to dysarthria or aphasia), a pragmatic clinical opinion of the neuropsychologist was requested. The patients were informed about the experiment, its inclusion and exclusion criteria, and the cognitive demand it implied. This additional evaluation was conducted before the participants were recruited and did not influence the allocation of the groups. After this first evaluation, carried out routinely by a physician and a neuropsychologist and/or speech therapist of the hospital, the patients were subjected to a second evaluation with a physiotherapist. The latter explained the experiment in detail and answered the questions of the patients. Signed informed consent was requested from the participants before the initial evaluation, following, throughout the study, the ethical principles of the Declaration of Helsinki for medical research in human beings [49]. This study was approved by the institutional board of the hospital and by the ethics committee of the university. Two physiotherapists were in charge of all the evaluations before and after the intervention and were blinded to the treatments. Only one of the physiotherapists with extensive experience in neurological physiotherapy and VR, performed all the interventions with VR. Both the evaluations and the treatments were conducted in one of the quiet rooms of the hospital, designed for individual treatments. Most of the selected patients were already receiving physiotherapy before the initial evaluation. 

### 2.2. Outcome Measures

To minimise the evaluation bias, we ensured that the initial and final evaluations of the same patient were carried out by the same physiotherapist. 

The primary variables were: the Fugl-Meyer scale (FM) specific for the LE [50], which was used to evaluate the motor function of the LE, with its subscales (amplitude/pain, sensitivity, motor evaluation, and balance) and, as functionality measures, the *Functional Ambulatory Category* (FAC) [51] and the Functional Independence Measure (FIM) [52]. The secondary variable was balanced, which was estimated using the Berg Balance Scale (BBS) [53]. Although many of the patients started from a high baseline score in the Trunk Control Test (TCT) [54], these data were also gathered since, despite their “ceiling effect”, they allowed us to evaluate patients in a more acute state [55]. 

The degree of satisfaction of the patients who received the VR treatment was measured through a questionnaire of 12 items, with a score of 1–5 each and a maximum of 60 points [56]. We also recorded the adverse effects and number of treatments lost throughout the study as an indicator of treatment safety. 

### 2.3. Interventions

All participants followed a treatment programme that consisted of 15 sessions of CP (1 h per day, 5 days a week). This baseline treatment was maintained since previous studies showed that the combined intervention of techniques with different CP approaches was significantly more effective than any treatment or control with placebo in the recovery of functional independence after a CVA [19]. Considering that it is an effective intervention that the patients already followed, we ethically decided not to modify it, adapting our study to the hospital routine and adding 1 more hour of CP to the control group in order to avoid differences in the results due to the greater intensity of the treatment that would have been derived from adding 1 h of VR in the experimental group. 

In the VR + CP group, the daily treatment consisted of 1 h of CP and 1 h of additional VR focused on the LE, whereas, in the CP group, the patients completed 2 h of CP. The physiotherapists were permanently present during the intervention session in both groups, adapting and progressing the physiotherapy programme, according to the motor capacities and needs of the patient. The equipment used for the VR therapy was a VRRS^R^ (Virtual Reality Rehabilitation System. Khymeia Group. Noventa Padovana, Italy), which included a computer, as the working station, connected to a 3D motion capturing system (*Polhemus Liberty*^TM^, Colchester, VT, USA), and a high-definition LCD projector, which was used to show the virtual scenarios in a large screen.

The therapy through VRRS involved the realisation of different types of motor tasks in which the patient had real objects as references (staircase steps, objects in high places, signs on the floor, etc.), interacting with a virtual scenario in which the movements of the LE were monitored using the motion capturing system, guiding the kinematic trajectories of movement in the different tasks. Moreover, a proprioceptive activity was performed with the aim of improving the stability and proprioception of the paretic LE by guiding the movement of the contralateral LE [28]. 

**Figure 1:**
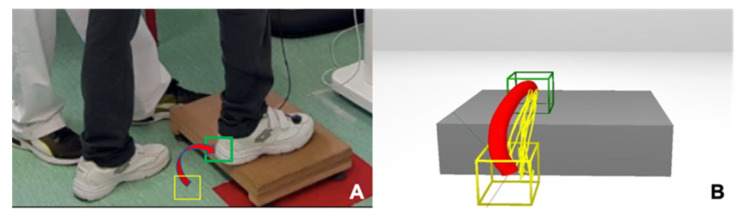
Representations of the task in the real and virtual stage. (**A**) Real scenario (raising the foot to a step); (**B**) virtual scenario that the patient could view on the screen. The red line represents the ideal trajectory, whereas the yellow lines indicate the patients’ trajectory in the different trials. Simultaneous visual-auditory feedback was provided to the patients during the execution of the movements. In addition, the physiotherapist provided feedback to the patients to enhance their performance.

For example (Figure 1), a simple movement, such as lifting a foot on a staircase step, was represented in the virtual scenario and represented by a virtual staircase step. The virtual scenario showed the correct movement trajectory of the foot climbing up the staircase step (red), previously recorded by the physiotherapist. Thus, the patient was asked to emulate (yellow) the correct movement trajectory shown on the screen, facilitating the perception of the patients and the correction of his/her movement errors through auditory/visual feedback, both during the realisation of the task and once it was finished, in order to visualise the obtained results (feedback of the performance and outcomes, respectively). The physiotherapist selected the characteristics and complexity of the motor tasks, modified the parameters of the software related to feedback (types of objects, trajectories, sounds, etc.), and applied a progression of difficulty based on the individual capacities of each patient. In this way, the patients were stimulated to activate different muscle groups of the LE with special difficulties, in order to execute increasingly complex tasks (each task was repeated until the correct performance was achieved; then, the number of repetitions was increased to consolidate the correct performance, before progressing in difficulty). The physiotherapist, in addition to managing the virtual environment to adapt it to the needs of each patient, guided them with verbal instructions when the patients encountered difficulties during the execution of the interactive exercise. In those cases in which the patient did not have a good initial trunk control, the progression of tasks started from the sitting position, progressing to walking whenever possible, and beginning with technical support if necessary. At the end of the intervention, the physiotherapist discussed with the patient the results obtained during the session in order to consolidate the motor learning achieved. 

The CP programme focused on 1 h/day of overall functional recovery of the patient (including the upper limb), based on mixed techniques with different approaches [57,58,59,60]. Patients assigned to the CP group received 1 more hour of specific LE rehabilitation consisting of stretching [61,62], passive, assisted, and active exercises in many directions in the lower extremity working space (e.g., hip joint flexion and extension, abduction and adduction, rotation internal and external, knee flexion, and extension). The exercises were performed in a sitting and standing position, and each of the training programs was customized to the motor abilities of the patients. Individual task-oriented exercises were selected for each patient according to their current mobility conditions (e.g., exercises for postural control in standing or sitting position instead of gait training). Then, the exercise program was progressively increased in terms of complexity by the physiotherapist in charge of the treatment (for example, going up and down stairs or exercises to improve dynamic balance), according to the results of the functional assessment. Thus, the exercises performed by the CP group patients were addressed to achieve the best functional skills for balance and autonomy of gait. Analytical work was conducted to improve the individualised deficits in each patient, with the final aim of re-educating the gait (stability on support, swing foot clearance, adequate preparation of the foot for initial contact, adequate step length, and energy conservation) [63]. To facilitate the re-learning of motor skills, the patients followed a series of motor tasks with increasing difficulty. Initially, we evaluated the movements in which the patient encountered difficulties in postural motor control, focusing on them. Then, the participants practised complex and combined movements, including the re-education of balance and gait, whenever possible. 

### 2.4. Statistical Analysis

The demographic and clinical variables were analysed through adequate statistical procedures in each case. To describe the total sample of patients, we used the means and standard deviations, and for the individual description of the 2 groups of patients, we used the median and the interquartile range (IR). 

Given the size of the groups and the absence of parametricity of the distributions in all variables (*Shapiro–Wilk* < 0.05), a non-parametric approach was applied in the analysis of the inter-and intra-group comparisons. The *Mann–Whitney* U-test was employed for the comparisons between the two groups prior to the intervention. Thus, we analysed whether the groups had significant differences in the scores of the scales at the beginning of the physiotherapy programme. For the intra-group comparisons, the Wilcoxon test was used, which allowed determining the scales in which the 2 groups of patients presented significant changes after the intervention. 

## 3. Results

**Figure 2:**
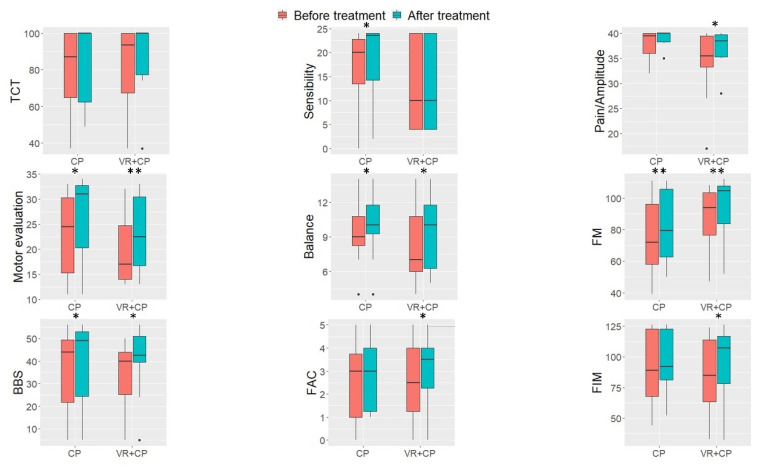
Boxplots of the scores obtained by the two groups of patients on each scale. TCT = Trunk Control Test; FM = Fugl-Meyer; BSS = Berg Balance Scale; FAC = Functional Ambulation Category; FIM = Functional Independence Measure. * *p* < 0.05, ** *p* < 0.01.

Group scores on each scale before and after the treatments were graphically represented as boxplots in Figure 2. For further information, individual scores on each of the scales before and after the treatment and statistical data were provided as supplementary material (See Tables S1 and S2). 

Between-group comparisons prior to the treatment revealed no statistical differences for any of the scales. Thus, they may be considered as having comparable initial clinical status. As for treatment effectiveness, overall increments can be observed in the scores of most of the scales and for both treatments (See Figure 2). 

In the CP group, scores in FM-subscales sensitivity, motor evaluation, and balance as well as in FM (total score), BBS, and FIM scales were significantly higher at the end of the intervention, as reported in the Wilcoxon tests. Improvements were also found in the remaining scales (TCT, amplitude/pain of FM and FAC) after the intervention, although comparisons did not reach the significant level. 

Moreover, the VR + CP group was found to significantly increase scores in all the scales, except for sensitivity and TCT. In addition, at the end of the intervention, this group displayed a high degree of satisfaction with the treatment (Median = 56.5, IR = 53.5–60).

## 4. Discussion

The present study compared the effects of an innovative modality of physiotherapeutic intervention (VR through reinforced feedback) with those obtained with conventional physiotherapeutic treatments in the functional recovery of the LE, balance, and gait after stroke. The results demonstrated the therapeutic effect of the treatment through VRRS, supporting the beneficial integration as a complement of CP. When combined, the CP and VR treatments increased functional gait and autonomy significantly more than the CP programme, as reported by scores in FIM and FAC scales (a specific scale of gait functionality).

Regarding motor function, both groups showed similar significantly better results in the final evaluation with respect to the initial evaluation. In FM, a significant improvement was obtained in the amplitude/pain subscale in the VR + CP group with respect to the CP group. One of the objectives of the intervention with VRRS was that patient completed the mobility ranges proposed in each of the exercises avoiding the use of synergies, obtaining arrival feedback. For example, in the seated position, we used sensors attached to the trunk to prevent it from moving while the LE was moving, thus achieving a more selective movement. This allowed the analysed approach to obtain positive results in joint range gain, with a reduction of pain. Future algometric tests could confirm this hypothesis. The sensitivity subscale improved significantly in the CP group but not in the VR + CP group. Nevertheless, through the VRRS treatment, the patient obtained proprioceptive information about his/her improvement in sensitivity (with the work on single leg stance of the paretic LE during the intervention with VRRS, conducting aerial trajectories with the contralateral LE), although the physiotherapist did not carry out specific work to improve superficial sensitivity with manual techniques, which may have influenced the results [21]. The intensification of work in this line in the CP group, by adding 1 h to this treatment, may have produced benefits. The different possibilities offered by CP and VR allow for an interesting combination of both modalities to add effects and achieve a functional improvement. The improvement in the balance subscale was significant in the two groups, in both FM and BBS; the previously mentioned work on single leg stance involved in both interventions (PC and VRRS) could be related to these positive results. In turn, the subscale *motor evaluation* presented significance in both groups. These significantly better results after the application of physiotherapy based on conventional methods and physiotherapy based on VRRS demonstrate that regardless of the functional improvement obtained in the VR + CP group, the effect of intensive physiotherapy provides a significant clinical improvement. The non-significance obtained in both groups in TCT may have been influenced by the ceiling effect of this scale on the sample since many of the patients started with the maximum score in the initial evaluation. However, the use of sensors in the trunk to monitor compensations, connected to those placed in the different segments of the LE while the patient was asked to perform the trajectories, may have improved the kinematics. Despite the existence of a correlation between the data obtained in FM and the kinematics [64], the specific evaluation of these parameters could reinforce these results in future studies. 

The positive results obtained in gait functionality, evaluated through FAC in the VR + CP group, are in line with those obtained by other authors who used this scale for evaluation after the application of a different specific VR system in the functional recovery of gait after stroke [65]. This research team also explored the positive effects of the VR intervention in terms of cortical reorganisation associated with motor recovery, since the intensive and repetitive use of the paretic LE (such as that involved in the tasks simulated through VR) induces positive effects on neuroplasticity and motor function [66]. Thus, movement re-learning implies a process of selection of motor actions to execute the required task [14,32]. Proposals such as the one presented in this study with VRRS allow promoting different paradigms (e.g., reinforced and supervised learning) that operate to encourage motor learning based on the received feedback. The adaptation of the type of feedback, auditory, and/or visual choice, the possibility to interact with virtual objects (which can be modified in size and position), and other options provided by the flexible software of this specific VR system have great advantages with respect to some VR systems based on commercial videogames, since the latter is not designed specifically for therapeutic purposes, and thus they present important limitations in terms of adaptation and progression of the feedback during the task [30]. The inherent combination of these specific VR systems with other systems (e.g., treadmills, robotics…, etc.) makes it difficult to discriminate the specific effect of VR. The system used in the present study did not have this limitation. 

Although gait speed is a fundamental result to assess gait functionality [67], in the present study, it was not possible to consider this parameter since a large number of participants started with a very low score in FAC, which means that they were not able to walk at the beginning of the intervention, at least without physical assistance, and thus a pre-post comparison could not be established, as the speeds could be influenced by the type of assistance provided. However, the possibility offered by the system to work with the patient in the sitting position allowed addressing patients who were initially poorly functional. This enables an earlier start in the treatment through VR and a progression from a more acute state of the different tasks. The possibilities of adaptation offered by the VRRS system were also very interesting since it makes it possible to place the different motion-capturing sensors in different body segments. This allowed recording kinetically ideal motion trajectories in the progression of the task, to which the patient approached progressively, guided by the feedback provided by the physiotherapist. The choice of tasks and trajectories were also fundamental for an optimal progression. This entails a research limitation since the interventions were personalised but could not be based on a closed and replicable protocol. A physiotherapist with years of clinical experience in neurological physiotherapy and extensive knowledge in VR conducted the intervention with VRRS to guarantee the most adequate programme for each patient. Completing and reinforcing learning, through the physiotherapeutic treatment based on VR, without the application of manual techniques but with continuous guidance, could be key in the leap that the patient needs toward greater autonomy. 

The degree of satisfaction obtained by the patients with the VRRS treatment was very high in most of them, which translates into a good acceptance of this novel treatment, in line with the results obtained in its previous application to the upper extremity [56]. Subject 3 was an exception, showing a surprising decrease in the score (30/60), with stabilisation in all the scales from pre to post and a worsening of trunk control, given the decrease in TCT score. Since trunk control is a capacity that patients acquire prematurely and usually improves in most patients, this finding could indicate a deterioration of the state of the patient unrelated to our intervention, which may have hindered the attainment of optimal results. The feasibility of the system was demonstrated, as no adverse effects related to its use were reported, and a high degree of satisfaction was obtained by the users. 

Next, we highlight some of the limitations of the present study. The aim of this pragmatic trial was to compare the effect of the VRRS treatment with standard treatment in a real clinical environment. Thus, a non-randomised technique was used in the allocation of patients. As a consequence of this, we observed that our patient groups differed in aspects such as post-stroke latency, type of stroke, and hemisphere affected between the groups. While it is certainly difficult to find comparable patients in all these factors, as well as in other demographic aspects, we acknowledge their possible influence on our results. One possibility would be to statistically control for their effects by introducing such variables into the statistical models. Unfortunately, this was not advisable in our study, given the small sample size. We can deduce that the duration and number of sessions used in the present study could be enough, since many authors have found that, with a smaller number of sessions and shorter duration, the obtained results were not as satisfactory as the ones reported by the literature using a larger number of sessions [34]. The evidence indicates that physiotherapy must include physical treatments that are clearly defined, well-described, and based on evidence, regardless of their historical or philosophical origin [68]. Despite the fact that controlled trials do not allow for a great description in this regard, previous studies without a comparison group have made it possible to thoroughly detail different options of tasks based on well-defined patterns of hemiparetic patients [28,31,69]. The limitations regarding the results of balance show the need for a platform of pressure capturing that can be coupled with sensors to provide feedback in this sense (it has been recently developed and used in patients). The multi-disciplinary work of the physiotherapist with other professionals poses an advance in the implementation of new systems and in the adaptation of the existing ones to the needs of physiotherapy. Demonstrating the efficacy of specific systems designed for the functional recovery of CVA patients with flexible software and hardware will make it possible for these systems to be more marketable, economic, and accessible to all our patients. 

## 5. Conclusions

The results reveal that the application of a VR treatment through increased feedback, combined with a CP programme, is more effective than the same amount of CP treatment in the functional improvement of the LE and gait after stroke. 

The treatment with VR is feasible and improves the joint range/pain, which could be related to this functional improvement. Although there were no significant changes in trunk control, since a large number of patients started with good initial control, the significant improvements obtained in both groups in balance and motor function reinforce the use of intensive physiotherapeutic treatment in CVA patients. 

The physiotherapeutic treatment with a specific VR system could be a complement to CP that would optimise the results. The role of the physiotherapist is essential in the implementation and adaptation of these new systems, with flexible software and hardware, to the individual needs of post-stroke patients. 

## Figures and Tables

**Table 1:** Demographic data. 1–10: VR + CP; 11–20: CP.

ID Patient	Sex	Age (Years)	Post-Stroke Months	Hemisphere of Stroke	Type of Stroke
1	Man	74	2.03	Left	Ischemic
2	Man	77	10	Left	Ischemic
3	Man	64	4.7	Right	Ischemic
4	Man	58	7.3	Left	Hemorrhagic
5	Man	50	2.8	Right	Hemorrhagic
6	Man	59	4.6	Right	Ischemic
7	Man	59	10	Left	Ischemic
8	Man	45	18.3	Right	Ischemic
9	Man	68	5.1	Left	Hemorrhagic
10	Man	73	4.1	Right	Hemorrhagic
11	Woman	76	6	Left	Ischemic
12	Woman	59	1	Left	Ischemic
13	Man	65	1.4	Left	Hemorrhagic
14	Woman	56	1.1	Left	Ischemic
15	Man	69	1.4	Left	Hemorrhagic
16	Woman	29	0.8	Left	Ischemic
17	Man	62	4	Left	Ischemic
18	Man	59	7.5	Left	Ischemic
19	Man	67	3	Left	Ischemic
20	Woman	80	1.5	Left	Ischemic

## Data Availability

Data supporting reported results can be found in Supplementary Materials.

## Supplementary Materials

The following are available online at https://www.mdpi.com/article/10.3390/jpm11111210/s1, Table S1: title: Individual results data of the clinical scales; Table S2: Median scores and interquartile ranges of the two groups of patients, before and after the intervention. Inter and intragroup contrasts.
