# Supplementary material for: Virtual Reality and Physiotherapy in Post-Stroke Functional Re-Education of the Lower Extremity: A Controlled Clinical Trial on a New Approach
Source: J Pers Med. 2021 Nov 16;11(11):1210. doi: 10.3390/jpm11111210 (PMC8622451; doi:10.3390/jpm11111210)
Supplement: Supplementary file 1 [file jpm-11-01210-s001.zip › jpm-1456559-supplementary.pdf]

Table S1. Individual results data of the clinical scales (1-10: *CP+VR*; 11-20: *CP*).

ID Patient		Fugl-Meyer	Functional Ambulation Category	Functional Independence Measure	Berg Balance Scale	Trunk Control Test	Degree of Satisfaction
1	pre	85	1	94	39	74	-
	post	99	2	100	47	100	60
2	pre	57	3	70	44	87	-
	post	62	3	70	48	100	55
3	pre	49	1	84	16	62	-
	post	50	1	84	17	49	30
4	pre	61	3	67	44	87	-
	post	73	3	81	53	100	52
5	pre	100	3	122	50	100	-
	post	108	4	122	53	100	58
6	pre	106	5	126	56	100	-
	post	111	5	126	56	100	60
7	pre	111	5	126	56	100	-
	post	111	5	126	56	100	60
8	pre	82	4	123	48	100	-
	post	86	4	123	50	100	54
9	pre	62	0	44	5	49	-
	post	65	1	52	15	49	59
10	pre	39	0	67	5	37	-
	post	57	1	81	5	50	55
11	pre	47	0	33	5	37	-
	post	52	0	32	5	37	-
12	pre	105	5	117	46	100	-
	post	109	4	117	56	100	-
13	pre	99	4	117	40	87	-
	post	103	4	117	44	87	-
14	pre	105	4	105	45	100	-
	post	108	5	113	53	100	-
15	pre	94	2	63	26	61	-
	post	107	3	73	41	74	-
16	pre	94	2	98	40	100	-
	post	106	4	122	48	100	-
17	pre	67	1	64	15	37	-
	post	73	1	78	24	74	-
18	pre	81	3	72	41	100	-
	post	89	3	101	41	100	-
19	pre	75	1	50	25	100	-
	post	82	2	79	39	100	-
20	pre	108	4	124	50	87	-
	post	112	4	126	52	100	-

Table S2: Median scores and interquartile ranges of the two groups of patients, before and after the intervention. Inter and intragroup contrasts.

Clinical scales		CP		CP+VR		Pre-Post Treatment comparisons[a]	
		Pre	Post	Pre	Post	CP	CP+VR
**Trunk Control Test**		87 (65-100)	100 (62.5 -100)	93.5 (67.5-100)	100 (77.3-100)	,1	,16
**FMLE**	a.Pain/ Ampl	39,5 (36-40)	40 (38.3-40)	35.5 (33.3-39.8´5)	38,50 (35.3-39.8)	,102	,02
	b.Sensibility	20,00 (13.5-22.8)	23,50 (14.3-24)	10,00 (4-24)	10,00 (4-24)	,03	1
	c. Motor evaluation	24,50 (15.3-30.3)	31,00 (20.3-32.8)	17,00 (14-24.8)	22.5 (16.8-30.5)	,02	,01
	d. Balance	9 (8.25-10.8)	10 (9.25-11.8)	7 (6-10.8)	10 (6.25-11.8)	,02	,03
	FM Total	72 (76.5-104)	79.5 (62.8-106)	79.8 (62.8-106)	105 (83.8-108)	,02	,02
**Berg Balance Scale**		44,00 (21.8-49.5)	49 (424.5- 53)	40 (25.3-44)	42.5 (39.5- 51)	,01	,01
**Functional Ambulation Category**		3 (1 – 3.75)	3 (1,25-4)	2.5 (1.25-4)	3,5 (2.25-4)	,15	,04
**Functional Independence Measure**		89 (67.8-123)	92 (81- 123)	85 (63.3-114)	107 (78.3-117)	,05	,03

[a]*Wilcoxon tests. FMLE (Fugl-Meyer Lower Extremity).*
